# Supplementary material for: Tracking the global reduction of marine traffic during the COVID-19 pandemic
Source: Nat Commun. 2021 Apr 27;12:2415. doi: 10.1038/s41467-021-22423-6 (PMC8079689; doi:10.1038/s41467-021-22423-6)
Supplement: Supplementary file 1 — Supplementary Information [file 41467_2021_22423_MOESM1_ESM.pdf]

Supplementary Information

Tracking the global reduction of marine traffic during the COVID-19 pandemic

David March*, Kristian Metcalfe, Joaquin Tintoré, Brendan J Godley

*Corresponding author: David March, email: D.March@exeter.ac.uk. Present address: Centre for Ecology and Conservation, College of Life & Environmental Sciences, University of Exeter, Cornwall Campus, Penryn TR10 9FE, United Kingdom.

Contents

Supplementary Tables (Supplementary Table 1)
Supplementary Figures (Supplementary Figures 1-9)
Supplementary Methods
Supplementary References

Supplementary Tables

Supplementary Table 1. Regression results for the six Linear Mixed Models (LMMs). Table shows the estimated beta coefficients and confidence intervals. Predictors: SIm (Stringency Index monthly median), Income (economic income levels, UM -upper middle-, LM -lower middle-, L -low-).

	All vessels	Cargo	Tanker	Passenger	Fishing	Other
Predictors	*Estimates (Conf. Int 95%)*	*Estimates (Conf. Int 95%)*	*Estimates (Conf. Int 95%)*	*Estimates (Conf. Int 95%)*	*Estimates (Conf. Int 95%)*	*Estimates (Conf. Int 95%)*
(Intercept)	4.78 ** (1.50 – 8.05)	0.00 (-2.38 – 2.38)	4.74 * (0.61 – 8.87)	9.34 (-2.98 – 21.66)	25.62 * (3.43 – 47.81)	13.61 *** (6.33 – 20.89)
SIm	-0.15 *** (-0.19 – -0.12)	-0.07 *** (-0.10 – -0.03)	-0.09 ** (-0.15 – -0.03)	-0.50 *** (-0.70 – -0.30)	-0.27 (-0.63 – 0.10)	-0.47 *** (-0.56 – -0.38)
Income [UM]	1.13 (-3.90 – 6.15)	0.34 (-3.31 – 3.98)	1.79 (-4.53 – 8.12)	14.59 (-6.35 – 35.53)	14.25 (-22.81 – 51.30)	6.31 (-4.95 – 17.57)
Income [LM]	0.11 (-4.91 – 5.13)	0.85 (-2.77 – 4.47)	-0.91 (-7.18 – 5.35)	8.50 (-13.24 – 30.23)	-6.98 (-44.85 – 30.89)	0.06 (-11.18 – 11.29)
Income [L]	-3.50 (-10.07 – 3.07)	3.14 (-1.58 – 7.85)	-2.49 (-10.63 – 5.65)	8.84 (-27.13 – 44.81)	-21.50 (-80.44 – 37.44)	-17.17 * (-31.71 – -2.63)
SIm * Income [UM]	-0.02 (-0.07 – 0.03)	-0.01 (-0.06 – 0.04)	-0.05 (-0.14 – 0.04)	-0.15 (-0.46 – 0.17)	-0.01 (-0.59 – 0.57)	-0.01 (-0.14 – 0.12)
SIm * Income [LM]	0.00 (-0.05 – 0.05)	-0.04 (-0.09 – 0.01)	-0.01 (-0.10 – 0.08)	0.21 (-0.13 – 0.54)	0.41 (-0.19 – 1.02)	0.15 * (0.02 – 0.29)
SIm * Income [L]	0.12 *** (0.05 – 0.19)	0.01 (-0.06 – 0.07)	0.11 (-0.01 – 0.22)	-0.05 (-0.57 – 0.46)	0.37 (-0.62 – 1.36)	0.41 *** (0.24 – 0.58)
**Random Effects**						
σ^2	87.31	71.49	247.15	2413.91	8168.96	525.59
τ_{00}	79.25 COUNTRY	29.12 COUNTRY	72.02 COUNTRY	458.25 COUNTRY	1098.77 COUNTRY	344.60 COUNTRY
N	124 COUNTRY	124 COUNTRY	124 COUNTRY	90 COUNTRY	90 COUNTRY	122 COUNTRY
Observations	741	741	741	537	537	729
Marginal R^2 / Conditional R^2	0.133 / 0.546	0.083 / 0.348	0.039 / 0.256	0.102 / 0.245	0.011 / 0.128	0.181 / 0.505

** p<0.05   ** p<0.01   *** p<0.001*

Supplementary Figures

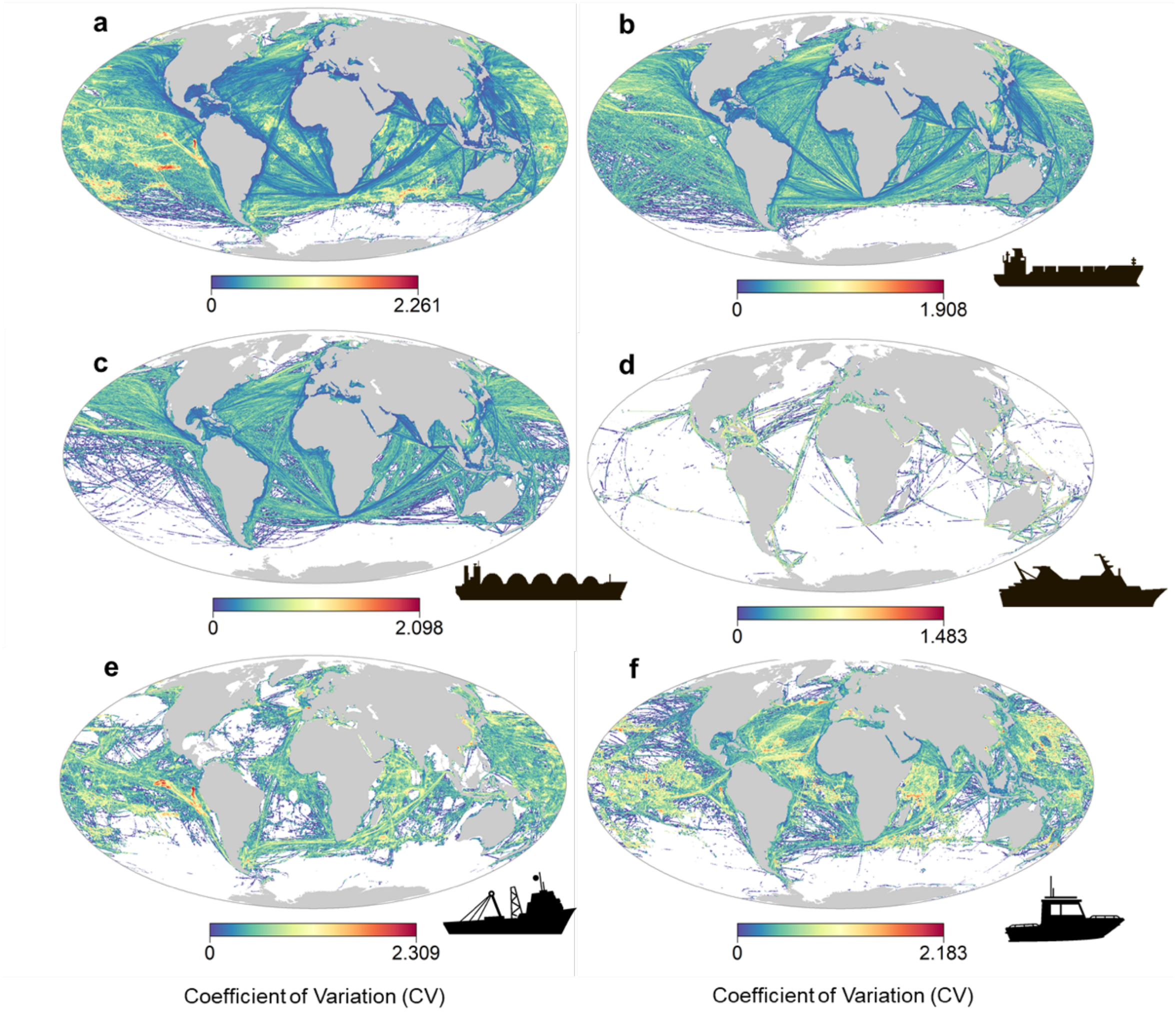

Supplementary Figure 1. Variability of marine traffic density during study period. Maps showing the Coefficient of Variation (CV) of monthly marine traffic density during the first half of 2020 (January - June). Vessel categories: (**a**) all vessels, (**b**) cargo, (**c**) tanker, (**d**) passenger, (**e**) fishing, and (**f**) other vessels. Higher values indicate areas of greatest variability in marine traffic density.

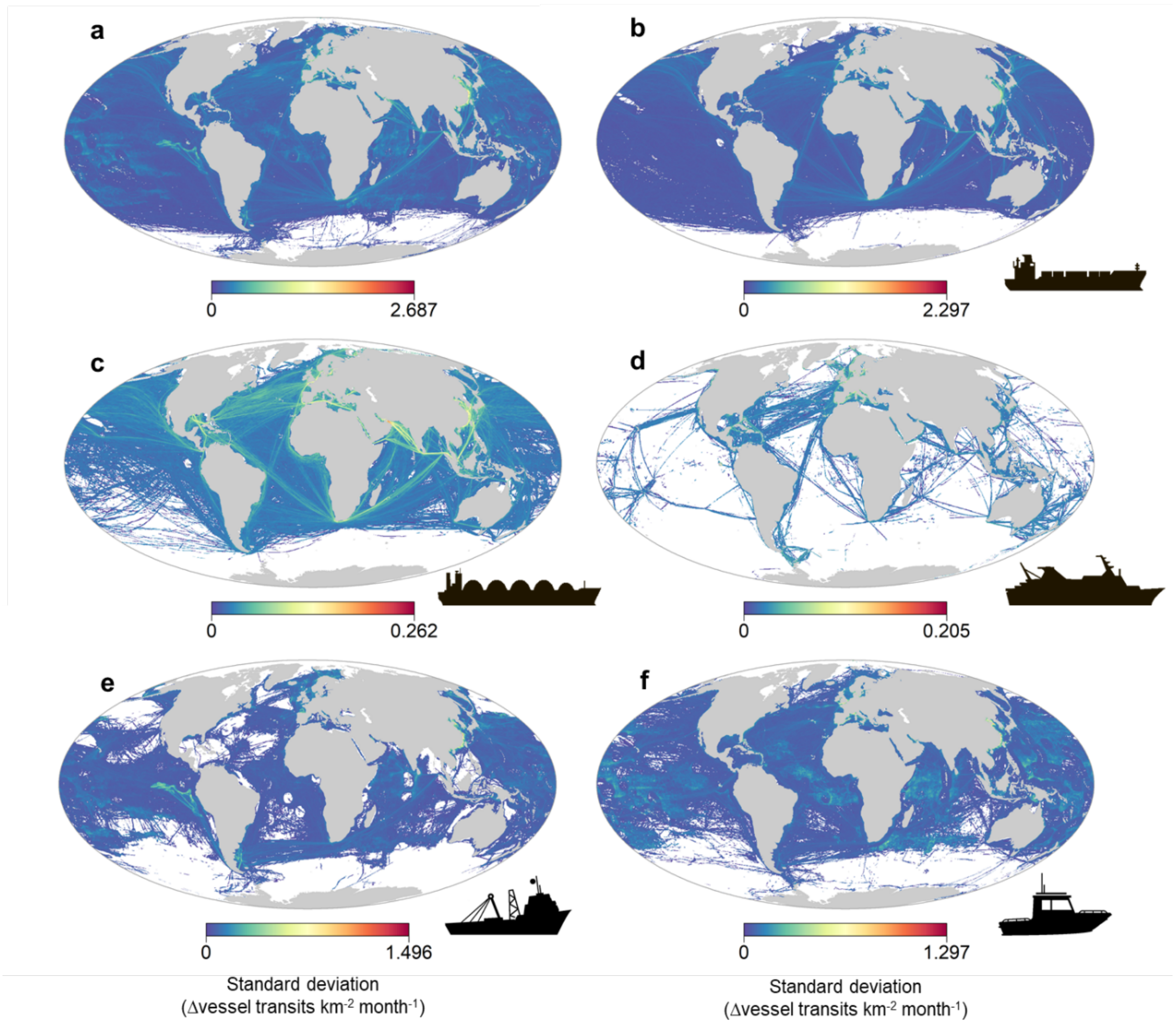

Supplementary Figure 2. Variability of global changes in vessel traffic density. Maps showing the standard deviation of the absolute difference in traffic density (number of vessel transits per square kilometre per month) between equivalent months (January – June) from 2020 and the reference year 2019. Absolute differences derived using cell-by-cell subtraction. Vessel categories: (**a**) all vessels, (**b**) cargo, (**c**) tanker, (**d**) passenger, (**e**) fishing, and (**f**) other vessels. Maps plotted on a square root transformed colour scale.

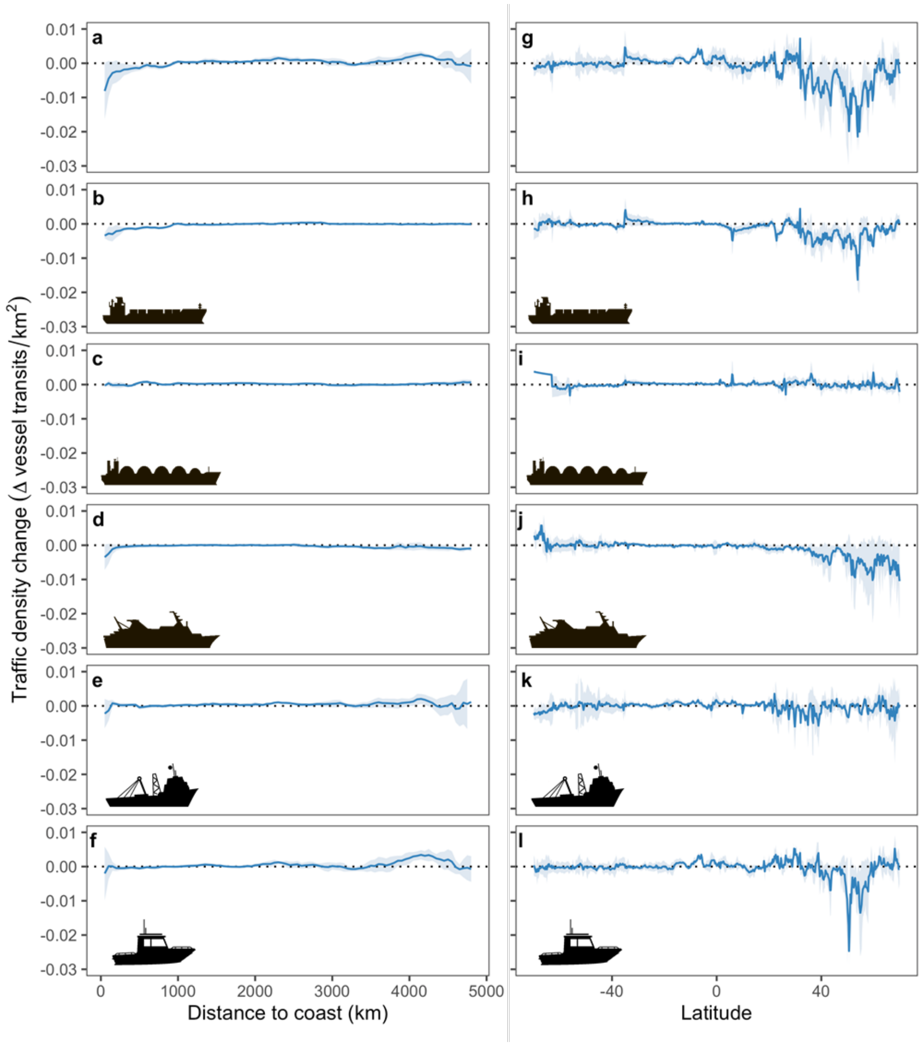

Supplementary Figure 3. Change in traffic density in relation to the distance to the coast (a-f) and the latitudinal gradient (g-l). Changes estimated by calculating the average of the absolute difference in traffic density (number of vessel transits per square kilometre and month) between equivalent months (January – June) from 2020 and the reference year 2019. Shaded area represents the standard deviation. Vessel categories: all vessels (**a, g**), cargo (**b, h**), tanker (**c, i**), passenger (**d, j**), fishing (**e, k**), and other vessels (**f, l**).

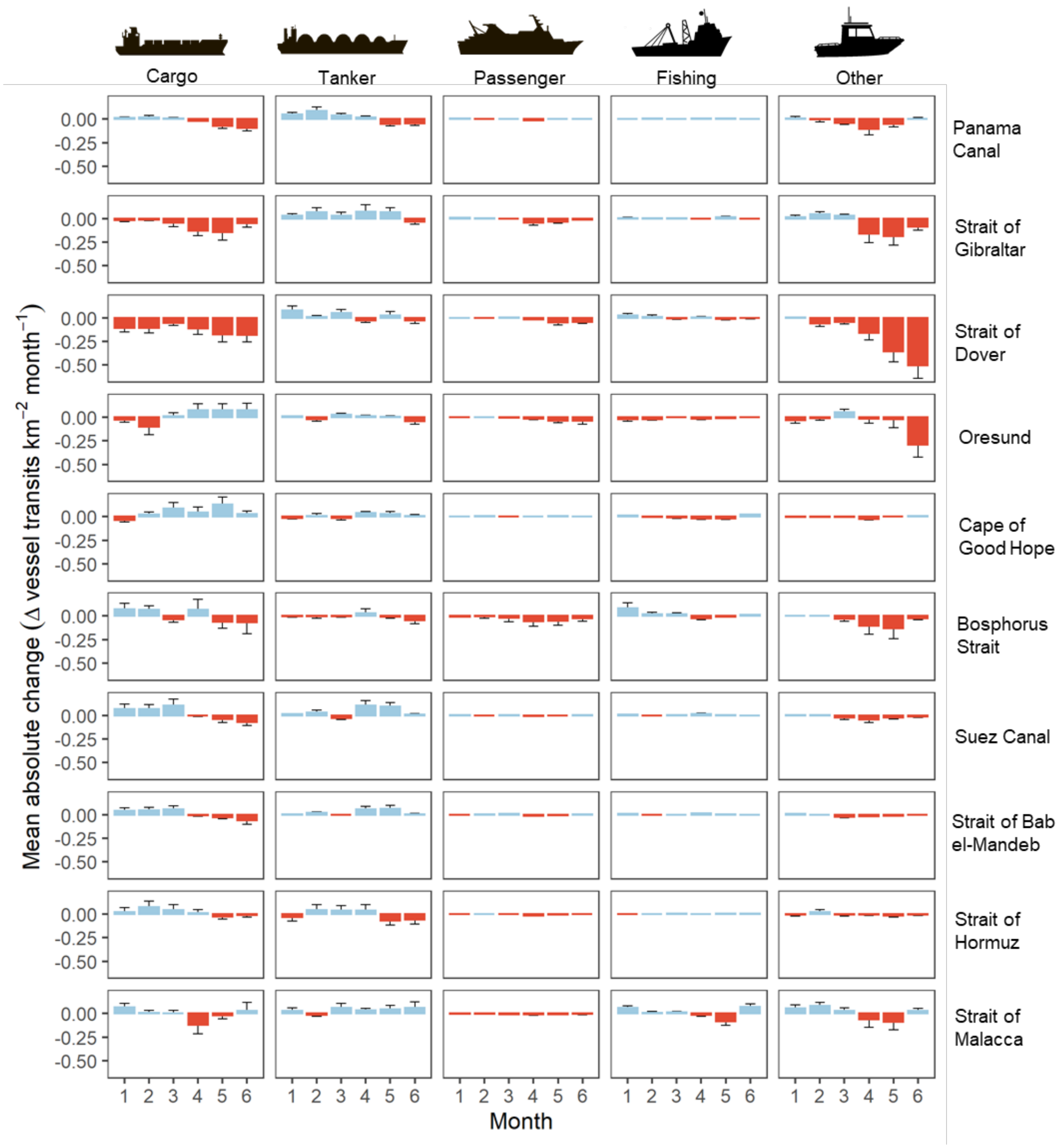

Supplementary Figure 4. Changes in marine traffic density in maritime chokepoints per vessel categories. Mean absolute change of marine traffic per month per chokepoint estimated within a 0.5 degrees radius from each chokepoint. Error bars represent standard deviation. Change estimates in comparison of monthly densities from equivalent month from reference year 2019. Refer to Figure 6 for a map with the location of maritime chokepoints. Months numbered January (1) - to June (6).

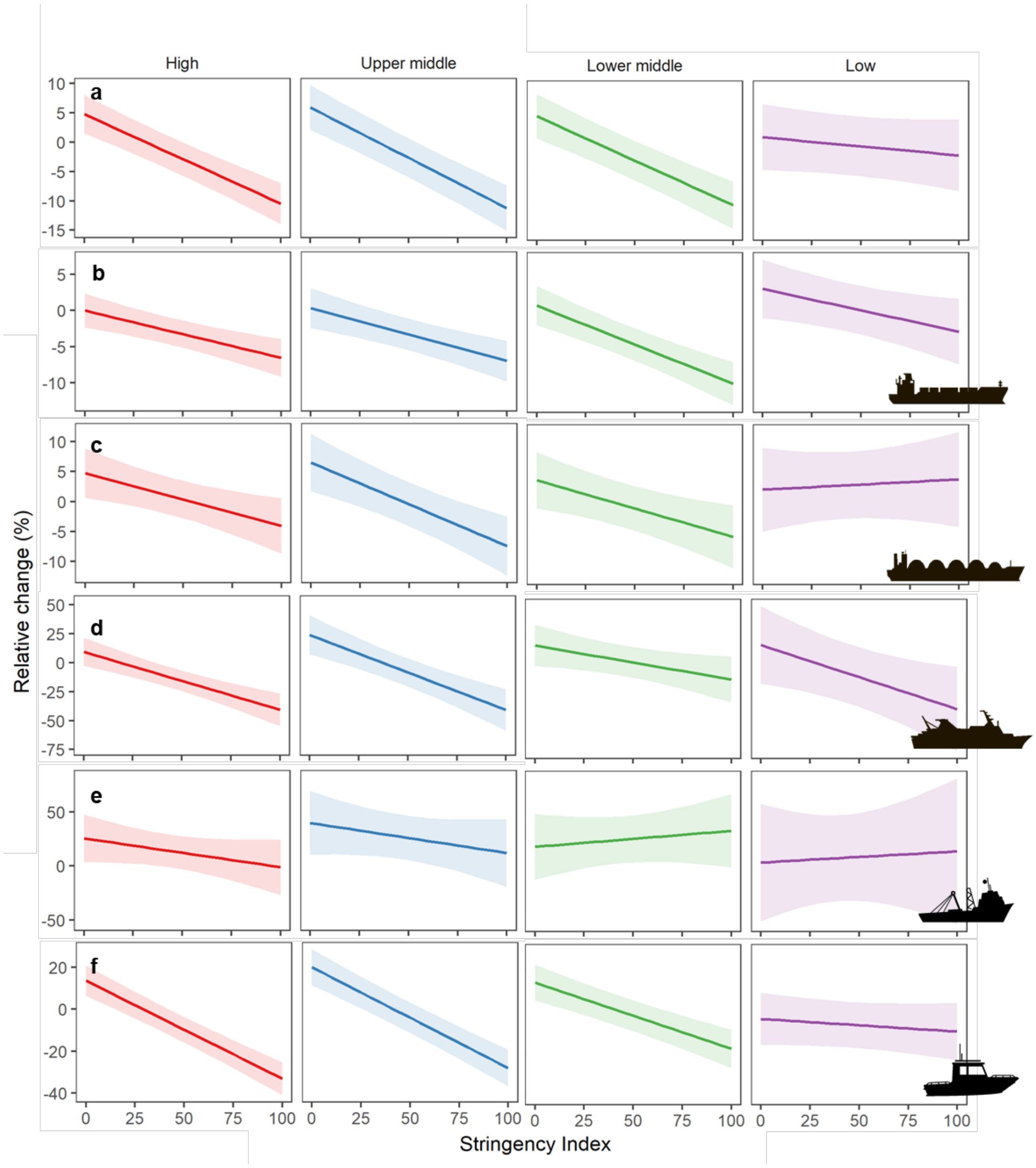

Supplementary Figure 5. Effect plot of the interaction in the mixed models between the Stringency Index and the Income groups for countries. Solid lines and shading represent the relative change of marine traffic density and 95% confidence interval, respectively. Each vessel category corresponds to an independent linear mixed-effect model (LMM): (**a**) all vessels, (**b**) cargo, (**c**) tanker, (**d**) passenger, (**e**) fishing, and (**f**) other vessels. Income levels were obtained from the World Bank and the Stringency Index (monthly median) from the Oxford COVID-19 Government Response Tracker (OxCGRT).

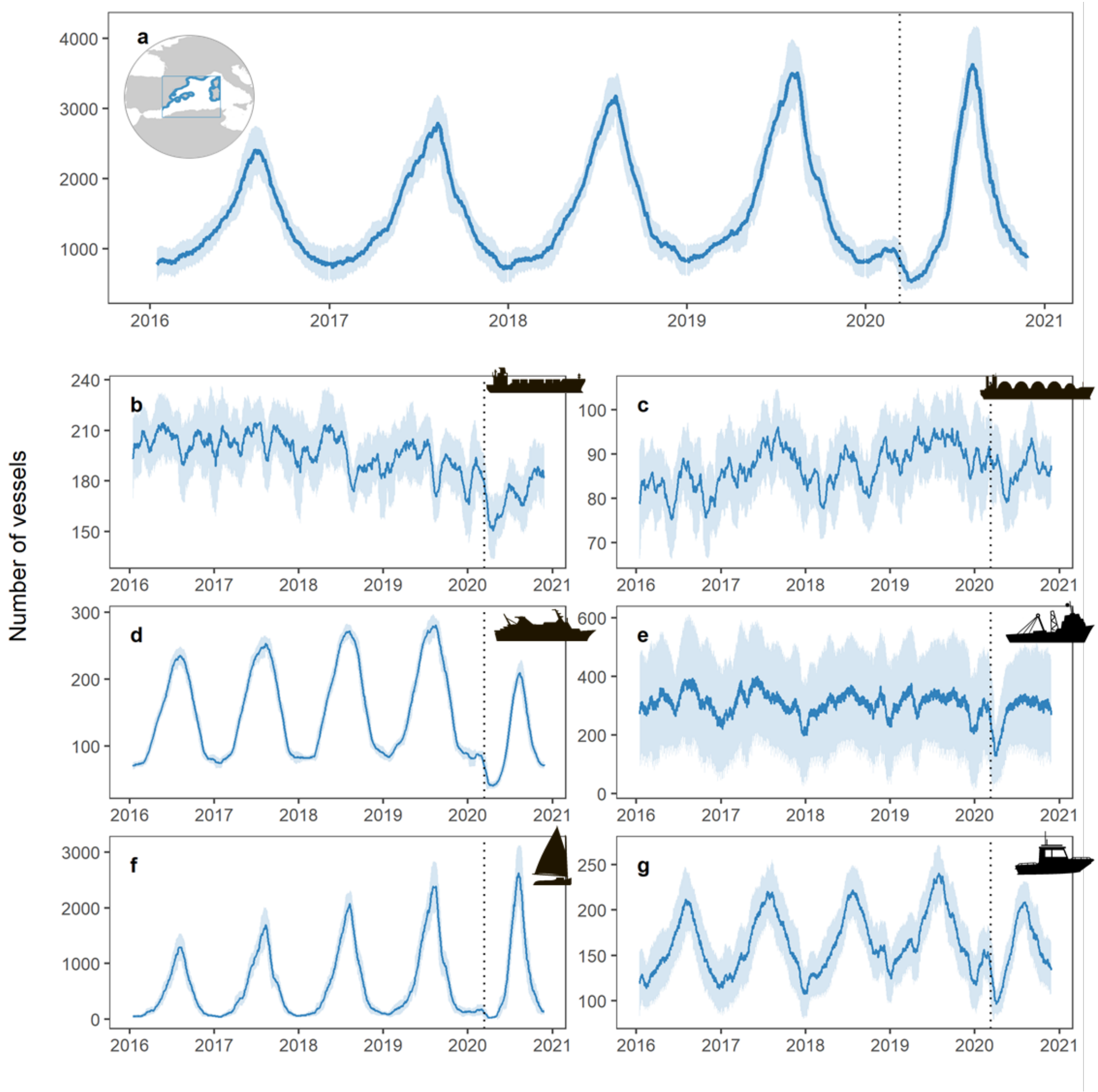

Supplementary Figure 6. Temporal variation of vessels in the Western Mediterranean between 2016 and 2020. Daily data of vessels underway within the coastal zone (24 nautical miles) of EU countries present in the study area (i.e., Spain, France, Italy) per vessel category: (**a**) All vessel types, (**b**) cargo, (**c**) tanker, (**d**) passenger, (**e**) fishing, (**f**) recreational, and (**g**) others. Daily estimates using 7-day moving average (from 1st January 2016 until 30th November 2020). Shaded area represents the standard deviation. Vertical dotted line represents the World Health Organization pandemic declaration on the 11th March 2020. Blue area in the map inset on part (a) represents the spatial extent of the regional Automatic Identification System dataset.

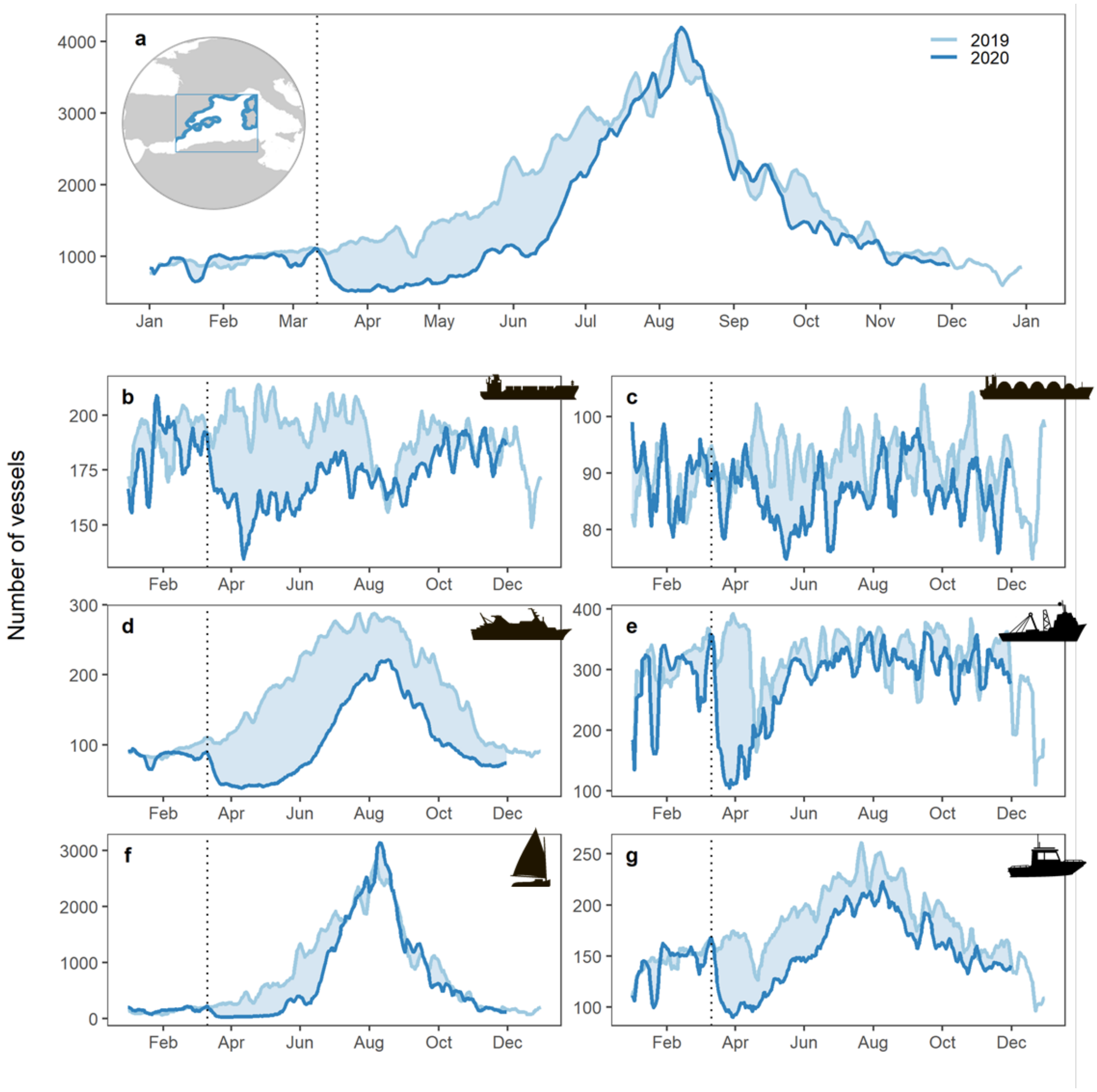

Supplementary Figure 7. Temporal variation of vessels in the Western Mediterranean during COVID-19. Daily data of vessels underway within the coastal zone (24 nautical miles) of EU countries present in the study area (i.e., Spain, France, Italy) per vessel category: (**a**) All vessel types, (**b**) cargo, (**c**) tanker, (**d**) passenger, (**e**) fishing, (**f**) recreational, and (**g**) others. Daily estimates using 7-day moving average. Shaded area represents the difference between 2019 and 2020 (from 1st January until 30th November). Vertical dotted line represents the World Health Organization pandemic declaration on the 11th March 2020. Blue area in the map inset on part (a) represents the spatial extent of the regional Automatic Identification System dataset.

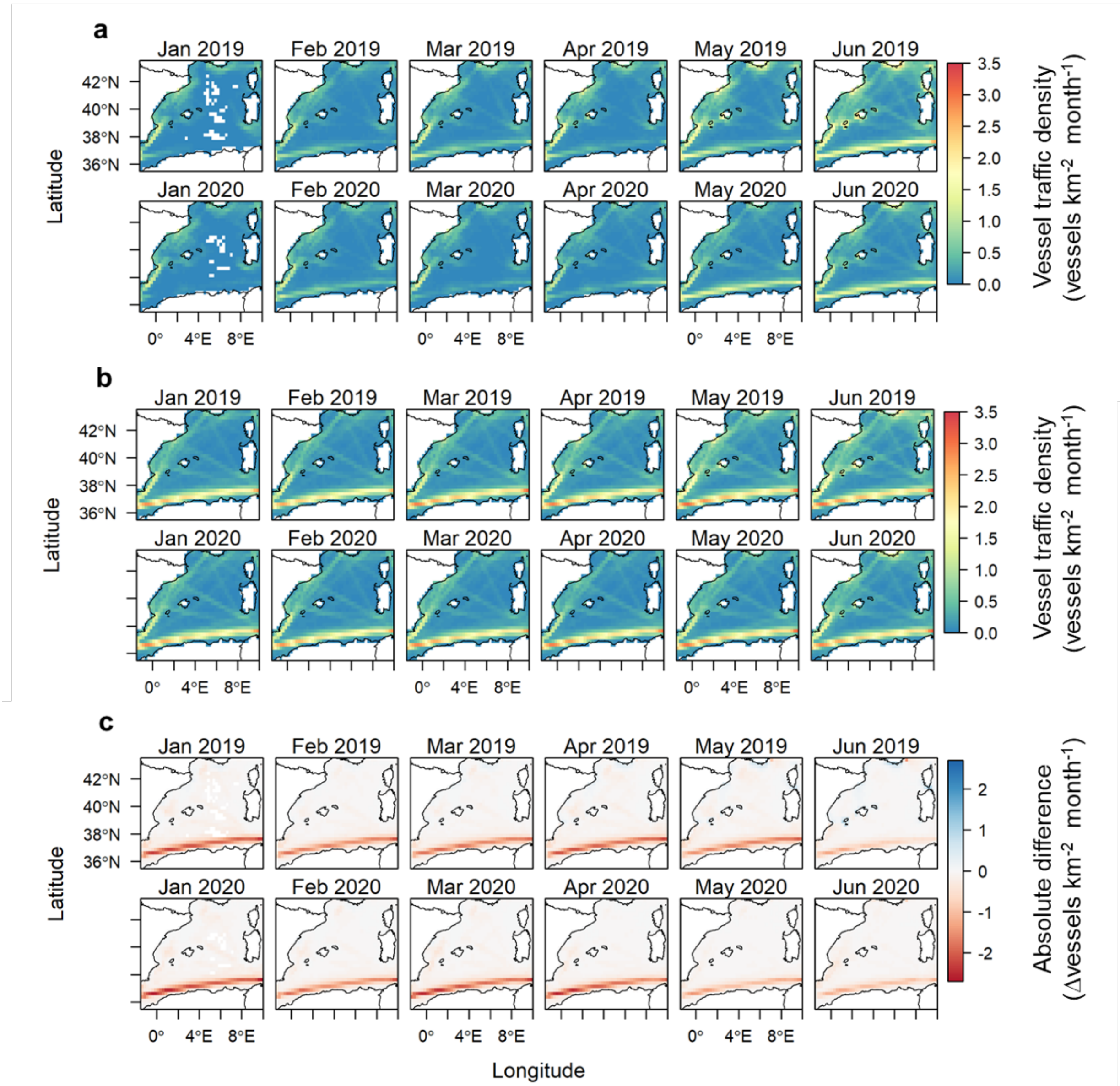

Supplementary Figure 8. Comparison between terrestrial (T-AIS) and satellite (S-AIS) Automatic Identification System data in the Western Mediterranean. (**a**) Monthly marine traffic density from T-AIS, (**b**) Monthly traffic density from S-AIS, (**c**) Absolute difference between T-AIS and S-AIS (i.e. negative values represent lower density for T-AIS). Density maps and difference consider all vessel types.

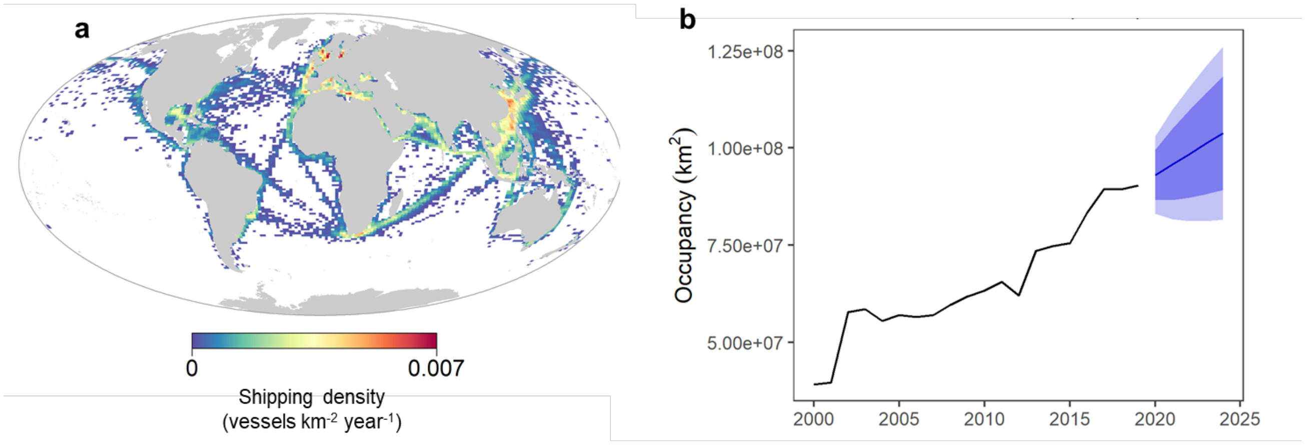

Supplementary Figure 9. Forecasts of global marine traffic occupancy. (**a**) Traffic density from altimetry data. (**b**) Forecasts of occupancy for 2020-2025, with confidence level for prediction intervals (80% and 95%)

Supplementary Methods

Post-processing S-AIS data

In order to remove inland water bodies from the satellite AIS dataset (S-AIS), grid cells from the Caspian Sea and with ≥95% land coverage were removed from the analysis. Land area coverage estimates were estimated based on the GSHHS shoreline database (version 2.3.7)[1], a high-resolution land mask that represented narrow straits (e.g. Suez Canal or Panama Canal). Further quality control procedures included the removal of ocean grid cells with vessel average speed values above a given threshold (i.e. 99th percentile) and small patches of isolated cells. Overall, we detected a total number of 1,143 patches across all processed months (n = 12). Each monthly map contained one large patch of interconnected grid cells (patch size > 2.5×10^8 km^2, n = 12). From the remaining patches, we filtered out patches of size < 769 km^2 (i.e. area of one grid cell in the equator). Visual inspection indicated that small patches with no variability in either the average speed or total number of vessels (i.e. standard deviation equals zero) were likely to be the result of spurious detections (e.g. linear patterns across same longitudes or latitudes), and were also filtered out. Finally, marine traffic density maps were converted to the Mollweide projection with a WGS84 datum as it is a single global projection that preserves geographic area and allows data transfer and analysis among operating systems and software.

Comparison between T-AIS and S-AIS in the Western Mediterranean

Terrestrial AIS (T-AIS) coverage was not homogenous in the Western Mediterranean due to a non-uniformly distribution of antennas (i.e. few antennas in North Africa, see www.marinetraffic.com). In order to assess the potential spatial and temporal bias in T-AIS we compared density estimates with the S-AIS dataset. First, we aggregated the T-AIS data into the same spatial (0.25 x 0.25 degrees) and temporal (monthly) resolutions than the S-AIS data, and reclassified vessels types into five categories (i.e. combined "recreational" with "other"). Then, we calculated the absolute difference between equivalent months across the study period (January - June in 2019 and 2020). Results show that S-AIS provides more information in areas with poor T-AIS coverage (Fig S8). We found a marked underestimation of traffic density by T-AIS in (1) areas with a lower density of antennas (i.e. north Africa), and (2) in areas furthest from the coast (e.g. ocean area between Balearic Islands and Sardinia). Moreover, we found higher underestimates during winter months, when AIS detection range could be reduced by adverse

metocean conditions. Overall, results align well with previous studies[2] and support our approach of restricting the analysis to EU coastal areas only.

Global estimates of the annual trends in shipping occupancy

To compare our estimates of changes in shipping occupancy (Fig 4b) with pre-COVID growth rates, we predicted occupancy for 2020 based on the annual trend from the previous 10 years. Given that no similar AIS dataset was available with an equivalent coverage, we used vessel density estimates from altimetry sensors[3]. Ship density estimates from altimetry were biased towards large vessels, which have higher radar reflectivity, and were acquired at coarser spatial resolution (i.e., 1 x 2 degrees). However, altimetry estimates provide a longer term series that can be combined with AIS data[4]. We derived annual occupancy maps following the same procedure used with S-AIS data (i.e. the extension of all cells with presence of marine traffic). Then, we used an autoregressive integrated moving average (ARIMA) model to forecast the expected occupancy in 2020-2025 based on the 2000-2019 period. The ARIMA model was performed using the *forecast* package with the auto.arima function, which implements an automatic stepwise model selection algorithm[5]. The selected model corresponded to the ARIMA (0, 1, 0), a random walk with drift, which provided an increase estimate of the occupancy for 2020 of 2.98% (Fig S9). While our approach assumes the same increase across the globe and do not distinguished between sectors, our estimate is similar to previous growth rates for some merchant vessels (e.g. 5% for roll-on/roll-off cargo ships, 2% for bulk carriers)[6].

Supplementary References

1. Wessel, P. & Smith, W. H. F. A global, self-consistent, hierarchical, high-resolution shoreline database. *J. Geophys. Res. Solid Earth* **101**, 8741–8743 (1996).
2. Holmes, S. *et al.* Where did the vessels go? An analysis of the EU fishing fleet gravitation between home ports, fishing grounds, landing ports and markets. *PLoS One* **15**, e0230494 (2020).
3. Tournadre, J. Anthropogenic pressure on the open ocean: The growth of ship traffic revealed by altimeter data analysis. *Geophys. Res. Lett.* **41**, 7924–7932 (2014).
4. Halpern, B. S. *et al.* Recent pace of change in human impact on the world's ocean. *Sci. Rep.* **9**, 11609 (2019).
5. Hyndman, R. J. & Khandakar, Y. Automatic Time Series Forecasting: The forecast Package for R. *J. Stat. Software; Vol 1, Issue 3* (2008).
6. Sardain, A., Sardain, E. & Leung, B. Global forecasts of shipping traffic and biological invasions to 2050. *Nat. Sustain.* **2**, 274–282 (2019).
